# JieZe-1 Alleviates HSV-2 Infection-Induced Genital Herpes in Balb/c Mice by Inhibiting Cell Apoptosis via Inducing Autophagy

**DOI:** 10.3389/fphar.2021.775521

**Published:** 2021-11-03

**Authors:** Qingqing Shao, Fan Wu, Tong Liu, Wenjia Wang, Tianli Liu, Ximing Jin, Lijun Xu, Yonggui Ma, Guangying Huang, Zhuo Chen

**Affiliations:** ^1^ Institute of Integrated Traditional Chinese and Western Medicine, Tongji Hospital, Tongji Medical College, Huazhong University of Science and Technology, Wuhan, China; ^2^ Department of Pharmacy, Tongji Hospital, Tongji Medical College, Huazhong University of Science and Technology, Wuhan, China; ^3^ Department of Integrated Traditional Chinese and Western Medicine, Tongji Hospital, Tongji Medical College, Huazhong University of Science and Technology, Wuhan, China

**Keywords:** JZ-1, HSV-2 infection, genital herpes, apoptosis, autophagy, PI3K/Akt/mTOR pathway

## Abstract

**Objectives:** Genital herpes (GH) is a common sexually transmitted disease mainly caused by herpes simplex virus 2 (HSV-2). JieZe-1 (JZ-1) is an in-hospital prescription that has been used in Tongji Hospital for many years to treat various lower female genital tract infectious diseases. Our previous study showed that JZ-1 can protect against HSV-2 infection *in vitro* by inducing autophagy. However, whether JZ-1 can protect against HSV-2 infection *in vivo*, and the underlying mechanisms involved still remain unclear. Therefore, this study was designed to address above questions.

**Methods:** 8-week-old female balb/c mice were injected intravaginally with HSV-2 to establish GH model. The symptom score, body weight, and histological examination were recorded to assess the animal model of HSV-2 infected and the therapeutic effect of JZ-1. Inflammatory response was determined by detecting inflammatory cells infiltration and local cytokines levels. After then, under autophagy inhibitor chloroquine application, we measured the levels of cell apoptosis and autophagy and investigated the relationship between enhanced autophagy and cell apoptosis. Next, the classic PI3K/Akt/mTOR axis was examined, and *in vitro* experiment was carried out for further verification.

**Results:** Our results showed that JZ-1 administration significantly reduces symptom score, increases weight gain and alleviates histological damage in HSV-2 infection-induced GH in balb/c mice. JZ-1 administration obviously ameliorates inflammatory responses with reduced T-lymphocytes, T helper cells, macrophages and neutrophils infiltration, and local IL-1β, IL-6, TNF-α and CCL2 levels. HSV-2 infection leads to massive cell apoptosis, which was also restored by JZ-1. Meanwhile, we found that HSV-2 infection blocks autophagic flux *in vivo* and JZ-1 administration induces autophagy. After chloroquine application, it was observed that the inhibition of autophagy is strongly associated with increased cell apoptosis, whereas the promotion of autophagy remarkedly decreases apoptosis. These results suggested that JZ-1 inhibits cell apoptosis in GH by inducing autophagy, which was further supported in later *in vitro* experiment. Additionally, PI3K/Akt/mTOR signaling pathway was also downregulated by JZ-1 administration.

**Conclusion:** Our data demonstrated that JZ-1 can alleviate HSV-2 infection-induced GH in balb/c mice by inhibiting cell apoptosis via inducing autophagy, and the underlying mechanisms may be associated with the inhibition of PI3K/Akt/mTOR pathway.

## Introduction

GH is a common sexually transmitted disease mainly caused by HSV-2, which brings a huge concern on personal and public health worldwide owing to its highly contagious. The prevalence of HSV-2 infection is strongly associated with increasing sexual activity, especially after adulthood (Fleming et al., 1997). It was estimated that the HSV-2 serum infection rate is as high as 20–80% in different populations of the world (Paz-Bailey et al., 2007). As the complete cure of HSV-2 infection is challenging, it will always lead to recurrent genital ulcers during the patients’ lifespan. Moreover, the mucosal destruction caused by genital ulcers provides easy access for the acquisition of human immunodeficiency virus (HIV), which increases the risk of sexually acquired HIV associated with HSV-2 infection (Corey et al., 2004). In addition, HSV-2 infection during pregnancy can lead to neonatal herpes as a result of vertical transmission (Kim and Lee, 2020). Currently, the main treatments for HSV-2 infection are nucleoside antiviral drugs, such as acyclovir, penciclovir and famciclovir. These antiviral drugs act by interfering with viral DNA polymerase, thereby inhibiting the replication of the viral genome (Kukhanova et al., 2014). However, these drugs are not very effective for skin lesion as they only shorten the recovery time of the lesions in 1–2 days in most cases, and the problem of drug resistance is also becoming increasingly prominent (Alvarez et al., 2020). Therefore, there is urgent need to explore new drugs that are more effective and long-lasting.

During HSV-2 infection, virus-driven cell death largely contributes to the development of clinical symptoms, like blister, ulcer and inflammation. Among which, autophagy and apoptosis were the most well-studied forms of cell death in virus infection. Autophagy is regarded as a cell homeostasis process that is maintained by fusion of autophagosomes with lysosomes to form autolysosomes. Increasing evidence revealed that viruses and intracellular bacteria can block autophagic flux in host cells to promote the progression of infection. Conversely, the activation of autophagy could be an effective approach to limit HSV-2 infection (Cavignac and Esclatine, 2010). Apoptosis is another kind of programmed cell death, which is characterized by cell shrinkage, formation of apoptotic bodies and nuclear fragmentation (Wyllie, 1997). It was found that apoptosis can be triggered by two distinct signaling pathways, the intrinsic and extrinsic pathways. The intrinsic apoptotic pathway is caused by a variety of intracellular stress conditions, including cytokine deprivation, DNA damage, oxidative stress, cytoplasmic Ca2+ overload and endoplasmic reticulum stress. And the extrinsic apoptotic pathway is elicited by extracellular stress stimulation that is sensed and triggered through activation of death receptors of the tumor necrosis factor (TNF) family, including TNF receptor 1 (TNF-R1), Fas and TNF-related apoptosis-inducing ligand (TRAIL) receptors (Galluzzi et al., 2012a; Zhou et al., 2017). Both pathways will converge on the activation of the cysteine-specific aspartic protease 3 (caspase-3) enzyme, causing proteolysis and ultimately leading to cell death (Galluzzi et al., 2012b). Consistent with autophagy, apoptosis is also closely related to virus infection. A number of studies have demonstrated that virus-mediated host cell apoptosis plays a crucial role in the pathogenesis of herpesviruses-related diseases, including HSV-2, varicella-zoster virus (VZV), murine cytomegalovirus (MCMV) and Epstein–Barr virus (EBV) (Yedowitz and Blaho, 2005; Stefanidou et al., 2013; Zhou et al., 2017). Autophagy usually precedes apoptosis in cells. In general, autophagy is a strategy for cells to adapt and respond to stress. Apoptosis program is activated when the stress exceeds the critical duration or intensity threshold acceptable to the cell (Marino et al., 2014). Therefore, promoting autophagy has an inhibitory role on cell apoptosis during virus infection, which has been confirmed by various investigations (Pei et al., 2016; Peng et al., 2016; Lu et al., 2020).

Traditional Chinese medicine (TCM) has a long history in the treatment of GH, and it was shown to exert good clinical effects both in short-term and long-term treatments with few side effects (Wang, 2008). In the theory of TCM, GH belongs to “sore of vulvae” and “leukorrheal disease”. The pathogenesis of this disease is summarized into three aspects: dampness, poison and deficiency. Dampness and heat are the main characteristics in the acute stage of GH, while in the recurrence and non-episodic stage, dampness, heat and deficiency exist simultaneously. Our research object JZ-1 is derived from the modification of Yihuang Tang, which was first described in Fu Qingzhu Nvke in the Qing Dynasty as a treatment for leukorrheal diseases. JZ-1 is composed of *Phellodendron chinense* Schneid. (Phellodendri Chinensis Cortex), *Ginkgo biloba* L. (Ginkgo Semen), *Solanum nigrum* L. (Solanum Nigrum), *Taraxacum mongolicum* Hand.-Mazz (Taraxaci Herba), *Thlaspi arvense* Linn. (Herba Patriniae), *Dictamnus dasycarpus* Turcz. (Dictamni Cortex), *Smilax glabra* Roxb. (Smilacis Glabrae Rhizoma), *Paeonia suffruticosa* Andr. (Moutan Cortex), *Mentha haplocalyx* Briq. (Menthae Haplocalycis Herba) and Borneolum Syntheticum. As an in-hospital preparation, JZ-1 has been used in Tongji Hospital for many years to treat various lower female genital tract infectious diseases including GH, and it has been proven effective. However, the underlying mechanisms of JZ-1 on GH are not fully elucidated.

Our previous study found that HSV-2 infection inhibited autophagy *in vitro*, and the antiviral effect of JZ-1 can be achieved by inducing autophagic flux (Shao et al., 2020). However, whether JZ-1 can protect against HSV-2 infection by inducing autophagic flux *in vivo*, and what is the relationship between enhanced autophagy and cell apoptosis under JZ-1 treatment. Given that, this work was designed to address above questions. In this study, we performed *in vivo* experiments to explore whether JZ-1 can promote autophagy in HSV-2 infected balb/c mice model and evaluated the potential connection between JZ-1-enhanced autophagy and cell apoptosis *in vivo* and *in vitro* by applying an autophagy flux inhibitor.

## Materials and Methods

### Herbal Preparation

JZ-1 consists of 10 traditional Chinese medicines, and the details of the herbal ingredients are shown in Table 1. All herbs were purchased from Hubei Shengdetang Chinese Herbal Pieces Co., Ltd. (Qianjiang, China). In brief, *Phellodendri Chinensis Cortex* was extracted twice using 70% ethanol. Except for Borneolum Syntheticum, the rest of herbs were boiled for 3 h, and then concentrated to 1.10∼1.20 g/ml gel under 60°C. After that, the above two extracts were combined, and then Borneolum Syntheticum was added to it. Finally, the solution was mixed and cooled to room temperature to obtain JZ-1 gel. For *in vitro* experiments, the process of JZ-1 preparation followed the same procedure but was not made to gel form. The quality control for JZ-1 has been evaluated by high-performance liquid chromatography (HPLC) fingerprinting in our recent publication (Duan et al., 2020).

**TABLE 1:** The composition of Jieze-1.

Latin name	English name	Chinese name	Used part	Weight (g)
*Phellodendron chinense* Schneid.	Phellodendri Chinensis Cortex	Hunagbo	Bark	10
*Ginkgo biloba* L.	Ginkgo Semen	Baiguo	Seed	10
*Solanum nigrum* L.	Solanum Nigrum	Longkui	Whole Plant	30
*Taraxacum mongolicum* Hand. - Mazz.	Taraxaci Herba	Pugongying	Whole Plant	15
*Thlaspi arvense* Linn.	Herba Patriniae	Baijiangcao	Whole Plant	30
*Dictamnus dasycarpus* Turcz.	Dictamni Cortex	Baixianpi	Velamen	10
*Smilax glabra* Roxb.	Smilacis Glabrae Rhizoma	Tufuling	Rhizome	15
*Paeonia suffruticosa* Andr.	Moutan Cortex	Mudanpi	Velamen	10
*Mentha haplocalyx* Briq.	Menthae Haplocalycis Herba	Bohe	Whole Plant	10
—	Borneolum Syntheticum	bingpian	—	0.3

### Animal Experiment and Drug Administration

8-week-old female balb/c mice were purchased from Beijing Vital River Laboratory Animal Technology Co., Ltd. and housed in Animal Biosafety Level 2 of Tongji Hospital under conditions of 12 h dark/light cycle, 60 ± 5% relative humidity and 20 ± 2°C environmental temperature. The animal experiment was reviewed and approved by the Animal Ethics Committee of Tongji Hospital, Tongji Medical College, Huazhong University of science and technology. After 1-week acclimation, balb/c mice were injected intramuscularly with progesterone (Zhejiang Xianju Pharmaceutical Co., Ltd.) daily (150 mg/kg) for 5 days, and then HSV-2 (2×10^4^∼2 × 10^6^ PFU/ml) was injected into the vagina of mice. Firstly, we explored the appropriate timepoint for drug intervention, thus eight timepoints (D1, D3, D5, D7, D9, D12 and D14) were set up after HSV-2 infection. The most typical timepoint was chosen for subsequent experiments by examining severity of HSV-2 infection.

Then the follow-up study was conducted based on the selected timepoint, and the following groups were included: normal group (blank gel), model group (HSV-2 + blank gel), JL group (HSV-2+0.5 mg/ml JZ-1 gel), JM group (HSV-2+1.5 mg/ml JZ-1 gel), JH group (HSV-2+2.5 mg/ml JZ-1 gel), acyclovir group (HSV-2+ acyclovir gel), chloroquine group (CQ intraperitoneal injection), chloroquine + model group (HSV-2+chloroquine), JZ-1+chloroquine + model group (HSV-2+CQ+2.5 mg/ml JZ-1 gel). Briefly, from day 1 on, mice in each group were injected with gel into the vagina twice a day until the end of the experiment. On day 6, all mice except mice of the normal group and chloroquine group were anesthetized, and then HSV-2 was injected into the vagina of mice. Chloroquine was injected intraperitoneally at a dose of 60 mg/kg every 3 days from the day before the model was established. On the end of the experiment, the mice were anaesthetized with 1% pentobarbital. After collecting blood sample, the mice were euthanized with CO_2_. After HSV-2 injection, the symptom score was determined daily according to Table 2.

**TABLE 2:** The details of symptom scoring.

Scores	Symptom
0	No symptoms
0.5	Increased vaginal discharge; mild vulvar redness
1	Redness and swelling of vulva
1.5	Redness and swelling of perineal
2	Obvious hair loss, small ulcers on the vulva and surrounding tissues (2 mm < d < 5 mm)
2.5	Obvious hair loss, small ulcers on the vulva and surrounding tissues (2 mm < d <5 mm) with purulence
3	Obvious hair loss, medium-sized ulcers on the vulva and surrounding tissues (5 mm < d < 10 mm)
3.5	Obvious hair loss, medium-sized ulcers on the vulva and surrounding tissues (5 mm < d < 10 mm) with purulence
4	Obvious hair loss, large ulcers on the vulva and surrounding tissues (d> 10 mm) with purulence
4.5	The hind limbs of the mouse are paralyzed and unable to move
5	The mouse died

### Chemicals and Antibodies

Chloroquine was purchased from Sigma-Aldrich, and bafilomycin A1 (Baf A1) was from Selleck. The following antibodies in immunoblotting and immunohistochemistry were used: anti-LC3B, anti-cleaved caspase-3, anti-Bcl-2, anti-p-Akt, anti-Akt, anti-p-PI3K, anti-PI3K, anti-mTOR, anti-p-mTOR and anti-gD were obtained from Cell Signaling Technology (Beverly, MA, United States). The antibodies for CD3 and MPO were from ABclonal Technology (Wuhan, China). Bax antibody was purchased from Proteintech (Wuhan, China). CD68 antibody was provided by Abcam (Cambridge, MA). CD4 antibody was obtained from Santa Cruz Biotechnology (Santa Cruz, CA). The antibody for β-actin was purchased from Servicebio Co.,Ltd. (Wuhan, China).

### Histological Examination

Fresh vulva tissues were fixed with 4% paraformaldehyde or placed instantly in liquid nitrogen and then transferred into −80°C refrigerator. Paraffin-embedded slides were used for hematoxylin-eosin (H&E) staining according to the standard protocol. The vulva histological images were captured by an Olympus BX51 system (Olympus, Japan).

### Immunohistochemistry Staining

Paraffin-embedded vulva slides were dewaxed by dimethylbenzene, polarized with descending concentrations of alcohol (75, 95, 100%, 5 min each), and rinsed with deionized water. After that, antigen retrieval was performed with a microwave. Endogenous peroxidase activity was blocked by incubating slides in 3% H_2_O_2_ at room temperature for 30 min. The 10% normal goat serum was used to block the slides for 1 h. Primary antibodies (anti-CD3, anti-CD4, anti-MPO, anti-CD68, anti-LC3B and anti-cleaved caspase-3) were applied overnight at 4°C followed by incubation of HRP-conjugated secondary antibody for 60 min at room temperature. The slides were visualized by DAB and counterstained with hematoxylin. Images were taken by an Olympus BX51 system and analyzed by ImageJ software (National Institutes of Health, United States).

### Quantitative Reverse Transcription PCR

Total RNA was extracted from the vulva tissue of the mice with TRIzol reagent (YEASEN, China) and reverse transcribed into cDNA using the Hieff First Strand cDNA Synthesis Super Mix (YEASEN, China) according to the manufacturer’s protocol. qRT-PCR was performed on a LightCycler^®^96 system (Roche Diagnostics, Mannheim, Germany) using Hieff qPCR SYBR Green Master Mix (YEASEN, China). The results were analyzed by the 2^−ΔΔCT^ method with β-actin as an internal control. The sequences of the primers used in this study are listed in Table 3.

**TABLE 3:** The primers used in this study.

Gene	Forward	Reverse
β-actin	CAC​CCA​GCA​CAA​TGA​AGA​TCA​AGA​T	CCA​GTT​TTT​AAA​TCC​TGA​GTC​AAG​C
gD	TCT​CCG​TCC​AGT​CGT​TTA​T	ATCCGAACGCAGCCCCGC
IL-1β	TCA​TTG​TGG​CTG​TGG​AGA​AG	AGG​CCA​CAG​GTA​TTT​TGT​CG
IL-6	CAA​TGA​GGA​GAC​TTG​CCT​GGT​G	TGG​CAT​TTG​TGG​TTG​GGT​CA
TNF-α	GCT​GCA​CTT​TGG​AGT​GAT​CG	ATG​AGG​TAC​AGG​CCC​TCT​GA
CCL2	CGC​ACT​AGG​TTT​GCC​GAG​TA	TGT​CTG​GAC​CCA​TTC​CTT​CTT​G

### Terminal Deoxynucleotidyl Transferase-Mediated dUTP Nick-End Labeling Staining

Paraffin-embedded vulva slides were routinely dewaxed and dehydrated, and then, the slides were immersed in 3% H_2_O_2_ methanol solution for 30 min at room temperature to terminate the peroxidase activity. Subsequently, the proteinase K working solution was supplemented and the slides were incubated at 37°C for 30 min. The slides were then immersed in 0.1% TrionX-100 and 0.1% sodium citrate solution and subjected to recovery at room temperature for 5 min. Then the TUNEL reaction mixture and the subsequent staining were proceeded according to the provided TUNEL kit instructions.

### Observations by Transmission Electron Microscopy

The fresh vulva tissues were collected and fixed overnight in cold glutaraldehyde fixative (2.5% in 0.1 mol/L cacodylate buffer, pH 7.4) and post-fixed in osmium tetroxide. After dehydration with a series of graded ethanol concentrations, the samples were rinsed with propylene oxide and impregnated with epoxy resins. Electron micrographs were taken by the transmission electron microscope.

### Cell Experiment

The human immortalized vaginal epithelial cell line (VK2/E6E7) was purchased from the American Type Culture Collection, and the Vero cell line was obtained from the Chinese Type Culture Collection. The VK2/E6E7 cells were cultured in Cn-TPR medium (CELLnTEC, Switzerland), and the Vero cells were cultured in Dulbecco’s modified Eagle medium (DMEM) containing 10% fetal bovine serum. The cells were grown at 37°C in a 5% CO_2_ atmosphere. According to our previous study, Baf A1 (100 nM) was cotreated with HSV-2 in the absence or presence of 5 mg/ml JZ-1 for the experiments for 24 h.

### Western Blot Analysis

Total protein was extracted from the vulva tissue of the mice or VK2/E6E7 cells with standard protocols, and the protein concentrations were quantified by the bicinchoninic acid (BCA) protein assay kit. Protein samples (40 μg/lane) were separated by SDS-PAGE (120 v, 90 min) and transferred to nitrocellulose (NC) membranes or polyvinylidene difluoride (PVDF) membranes. After blocking with 5% BSA powder with ultrapure water for 1 h, the membranes were incubated overnight with primary antibodies (anti-LC3B, anti-cleaved caspase-3, anti-Bcl-2, anti-bax, anti-p-Akt, anti-Akt, anti-p-PI3K, anti-PI3K, anti-mTOR, anti-gD and anti-β-actin) at 4°C. After washing three times with TBST for 10 min each, the membranes were incubated with secondary antibodies for 1 h at room temperature, washed another three times, and then visualized by an Odyssey Infrared Imaging system (LI-COR Biosciences, United States). The band densities were quantified by ImageJ (National Institutes of Health, United States).

### Statistical Analysis

All data are presented as means ± SEM. Statistical analyses were performed followed this rule: Firstly, the normality of data is tested by Shapiro-Wilk test. Secondly, data that fits the normal distribution are tested for homogeneity of variance via one-way ANOVA. Finally, Tukey’s multiple comparisons test can be performed to compare multiple groups only if there is no significant variance inhomogeneity between groups. Mann-Whitney U test should be used as an alternative post hoc test when the data show non-normality or variance inhomogeneity. Statistics were analyzed using the GraphPad Prism 8 software, and *p* < 0.05 was considered as statistically significant.

## Results

### Balb/c Mice have the Most Severe Symptoms on the 9th Day After HSV-2 Infection

**FIGURE 1:**
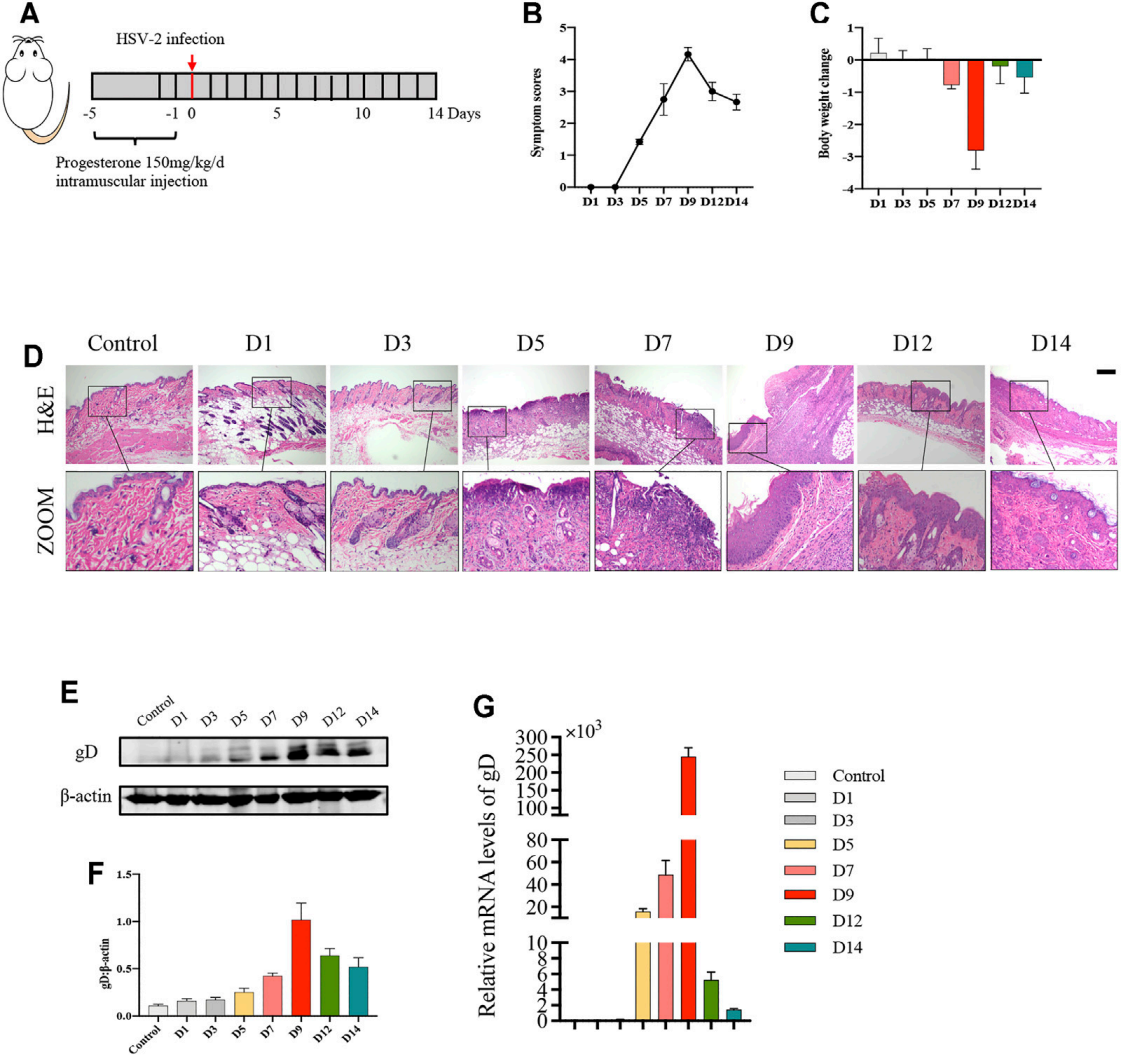
Balb/c mice have the most severe symptoms on the 9th day after HSV-2 infection. **(A)** The protocol of timepoint exploring for animal experiment. **(B)** Symptom scores of different groups in each timepoint. **(C)** Body weight change of different groups in each timepoint (compared with body weight of mice before treatment. **(D)** Pictures of the vulva of balb/c mice. **(E)** Representative western blots for gD protein expressions in vulva. **(F)** The quantification of gD western blots. **(G)** The mRNA levels of gD. All data are presented as means ± SEM. *p* < 0.05 (*), *p* < 0.01 (**), and *p* < 0.001 (***); (*n* = 6).

To determine the appropriate timepoint for the degree of genital herpes caused by HSV-2 infection in balb/c mice, we set 8 timepoints after HSV-2 infection (D1, D3, D5, D7, D9, D12 and D14) (Figure 1A). The results showed that on the 9th day after vaginal administration of HSV-2, the mice had the highest vulvar symptom score and the most significant weight loss (Figures 1B,C). The results of H&E staining also showed that the structure was disordered and the degree of inflammatory cell infiltration was the heaviest on the 9th day (Figure 1D). In addition, the expression level of vulvar virus protein gD was the highest on the 9th day (Figures 1E–G). Therefore, the 9th day after vaginal administration of HSV-2 was selected as the timepoint for the follow-up experiments.

### JZ-1 Administration Significantly Alleviates HSV-2-Induced GH in Balb/c Mice

**FIGURE 2:**
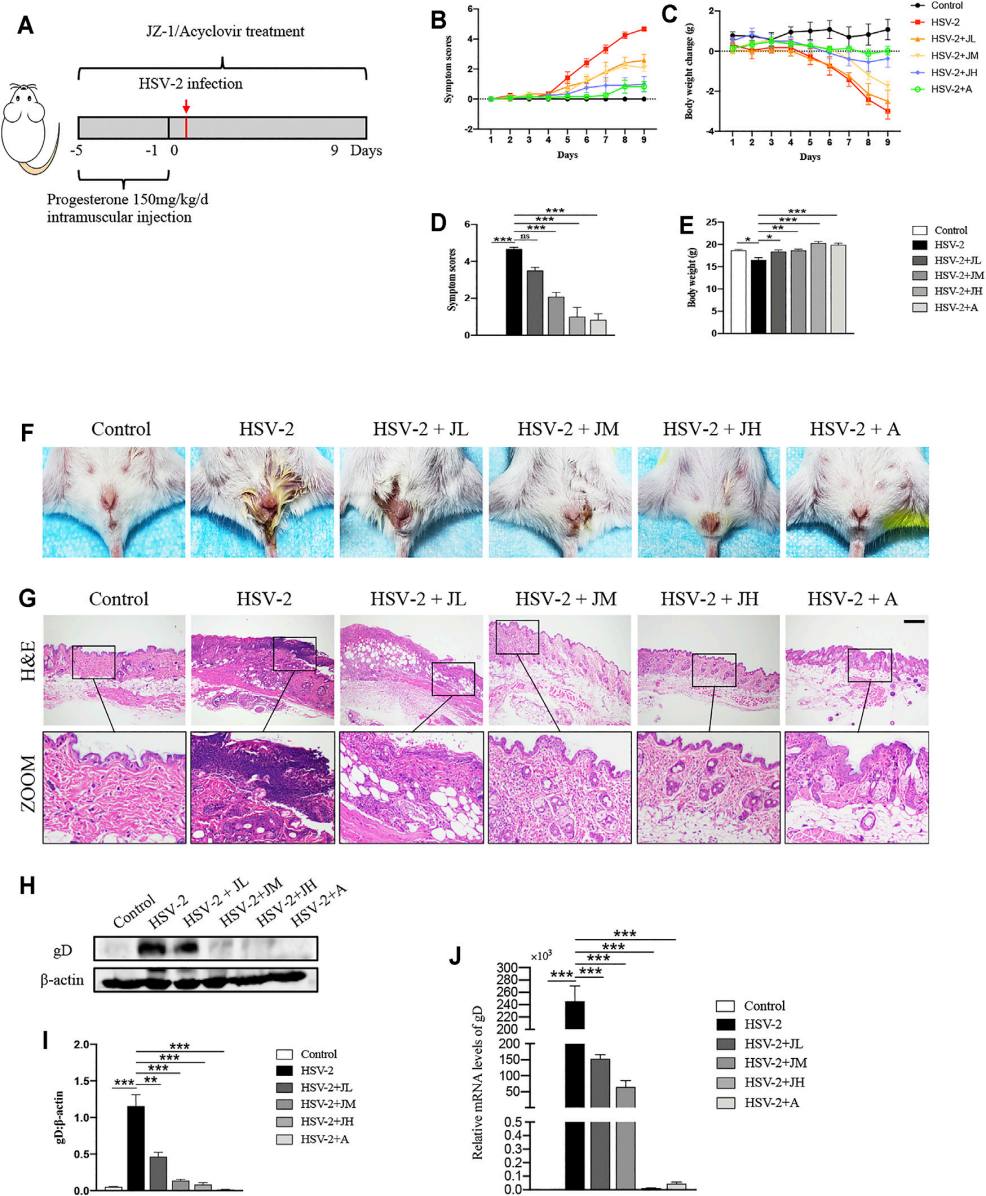
JZ-1 administration significantly alleviates HSV-2-induced GH in balb/c mice. **(A)** The protocol of animal experiment. **(B)** Symptom scores of different groups over time. **(C)** Body weight change of different groups over time (compared with body weight of mice before treatment. **(D)** Symptom scores were recorded at the ending of experiment. **(E)** Body weight was recorded at the ending of experiment. **(F)** Pictures of the vulva of balb/c mice. **(G)** Representative liver H&E staining of different groups. Scale bar, 200 μm. **(H)** Representative western blots for gD protein expressions in vulva. **(I)** The quantification of gD western blots. **(J)** The mRNA levels of gD in vulva. All data are presented as means ± SEM. *p* < 0.05 (*), *p* < 0.01 (**), and *p* < 0.001 (***); (*n* = 6).

In order to clarify the anti-HSV-2 effect of JZ-1, three doses of JZ-1 were used in HSV-2 infected balb/c mice, and acyclovir was chosen as the positive control drug (Figure 2A). Compared to the model group, JH administration exerted a significant symptom-improving and weight-regaining effect similar to acyclovir, and JM is less effective in improving symptoms and weight than JH. While JL administration had a trend of symptom-improving and weight-regaining although it was not statistically significant (Figures 2B–F). The results of H&E staining showed that the vulva structure of the mice in model group was disordered, the epidermis was hyperplasia, and there were a large number of inflammatory cell infiltration, which was largely restored by JZ-1 administration, especially JH administration (Figure 2G).

HSV-2 glycoprotein D (gD) is a receptor binding protein required by most alpha herpes viruses including HSV-2 and thus plays a vital role in the spread of the virus (Connolly et al., 2021). The trend of the expression levels of gD was also consistent with the histological results (Figures 2H–J). Collectively, these data suggested that JZ-1 administration significantly alleviates HSV-2-induced GH. Due to JH showed the best therapeutic effects, hence, we choose JH group as the representative JZ-1 intervention group in later mechanism investigations.

### JZ-1 Administration Inhibits Inflammatory Response in HSV-2-Induced GH

**FIGURE 3:**
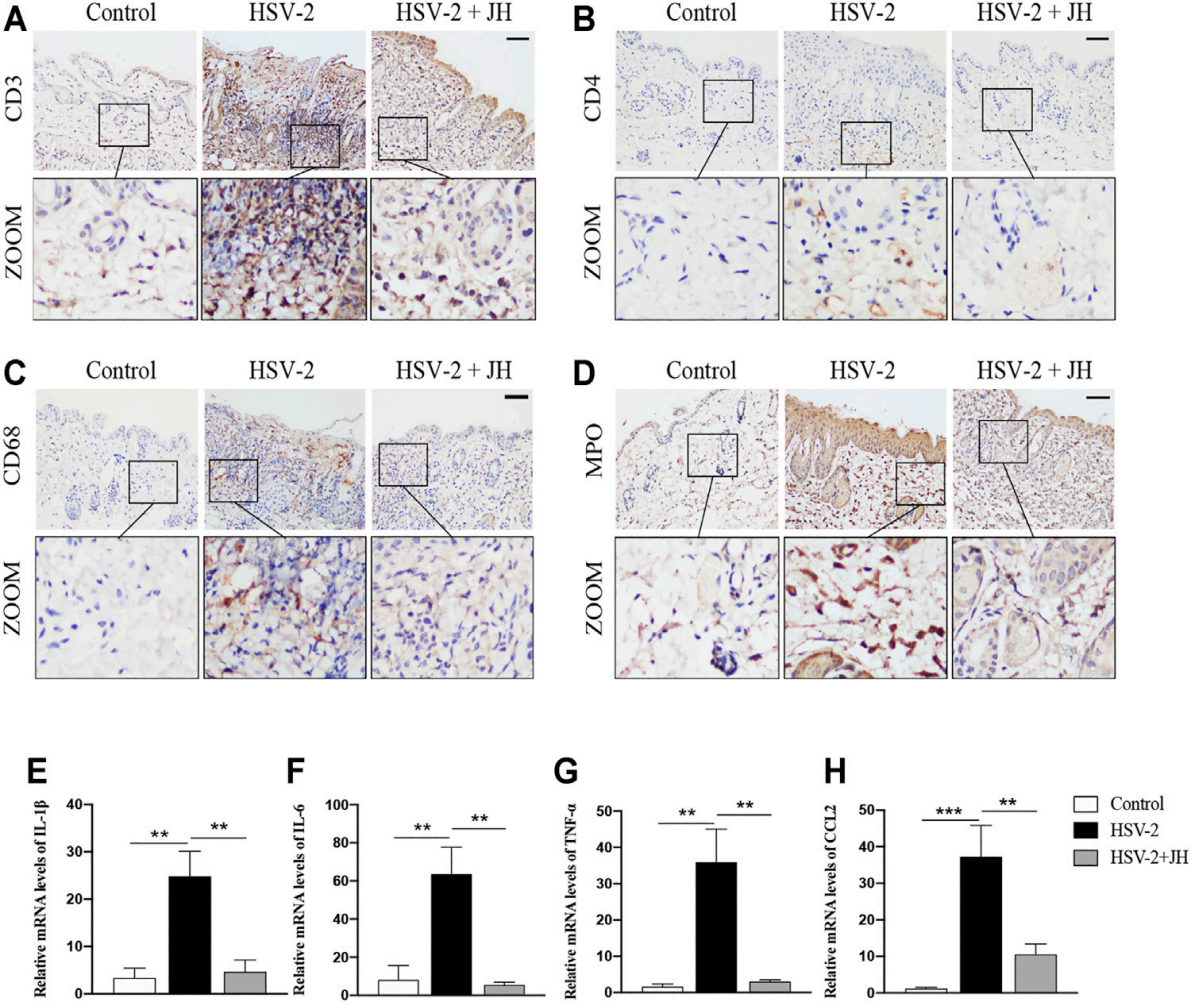
JZ-1 administration inhibits inflammatory response in HSV-2-induced GH. **(A–D)** Representative immunohistochemistry staining for CD3, CD4, CD68 and MPO in vulva of different groups. Scale bar, 100 μm. **(E–H)** The mRNA levels of IL-1β, IL-6, TNF-α and CCL2 in vulva of different groups. All data are presented as means ± SEM. *p* < 0.05 (*); (*n* = 6).

To investigate the effects of JZ-1 on inflammatory response in GH, we detected the levels of inflammatory cell infiltration and pro-inflammatory cytokines expression. As shown in Figures 3A–D, the results of IHC staining showed that, compared with control group, T-lymphocytes (CD3-positive), T helper cells (CD4-positive), macrophages (CD68-positive) and neutrophils (MPO-positive) in model group were significantly increased. Compared with model group, JZ-1 significantly decreased the inflammatory cell infiltration. Also, we examined the mRNA levels of pro-inflammatory cytokines, including IL-1β, IL-6, TNF-α and chemokines CCL2, using qRT-PCR assays. Consistent with the results of IHC staining, the mRNA levels of pro-inflammatory cytokines increased obviously in the HSV-2 infected balb/c mice, whereas those increased pro-inflammatory cytokines were largely restored by JH administration (Figures 3E–H). Altogether, these results indicated that JZ-1 administration inhibits inflammatory response in HSV-2-induced GH.

### JZ-1 Administration Suppresses Cell Apoptosis in HSV-2-Induced GH

**FIGURE 4:**
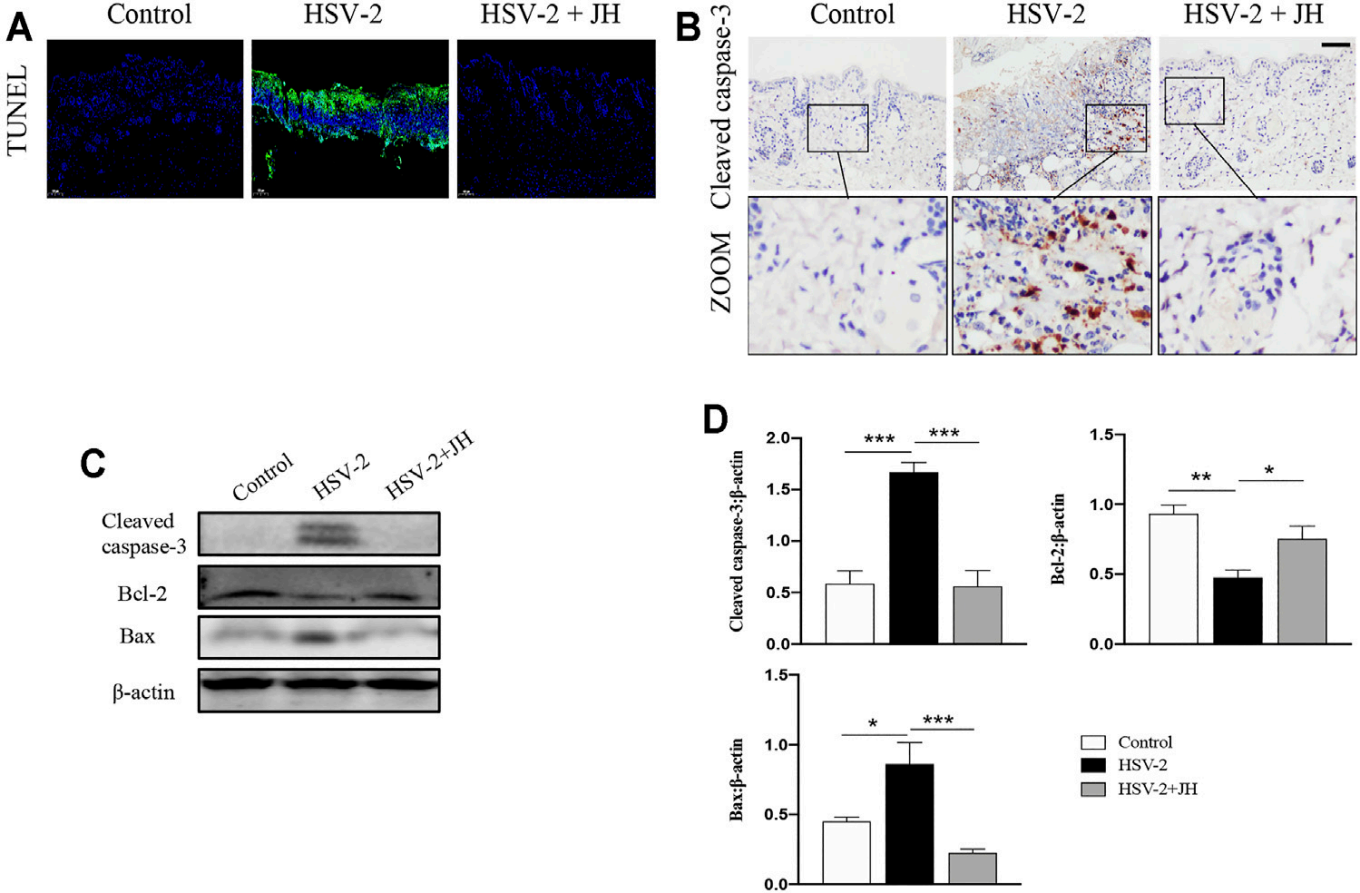
JZ-1 administration suppresses cell apoptosis in HSV-2-induced GH. **(A)** Representative TUNEL staining in vulva. **(B)** Representative immunohistochemistry staining for cleaved caspase-3 in vulva. **(C)** Representative western blots for cleaved caspase-3 and Bax in vulva. **(D)** The quantification of cleaved caspase-3 and Bax western blots. All data are presented as means ± SEM. *p* < 0.01 (**), and *p* < 0.001 (***); (*n* = 6).

It has been proved that cell apoptosis plays a key role in the development and progression of herpesviruses-related diseases. To determine whether JZ-1 could affect cell apoptosis in HSV-2-induced GH, TUNEL staining was applied to observe apoptotic cells. TUNEL staining results revealed that cell apoptosis of model group was remarkedly increased compared to that of control group (green area), and JZ-1 administration suppressed excessive cell apoptosis compared to model group (Figure 4A). Meanwhile, cell apoptosis was also detected by apoptotic maker cleaved caspase-3 IHC staining, and the results were similar to TUNEL staining (Figure 4B). Next, we tested the protein expression of cleaved caspase-3, Bcl-2 (a well-known anti-apoptotic protein) and Bax (a well-known pro-apoptotic protein) by western blot. As expected, compared to model group, JH treatment brought a significant reduction in cleaved caspase-3 and Bax expressions, and increased the expression of the anti-apoptotic protein Bcl-2 (Figures 4C,D). Therefore, our findings demonstrated that JZ-1 administration suppresses cell apoptosis in HSV-2-induced GH.

### HSV-2 Infection Leads to a Blockage of Autophagic Flux *in vivo* and JZ-1 Administration Induces Autophagy in HSV-2-Induced GH

**FIGURE 5:**
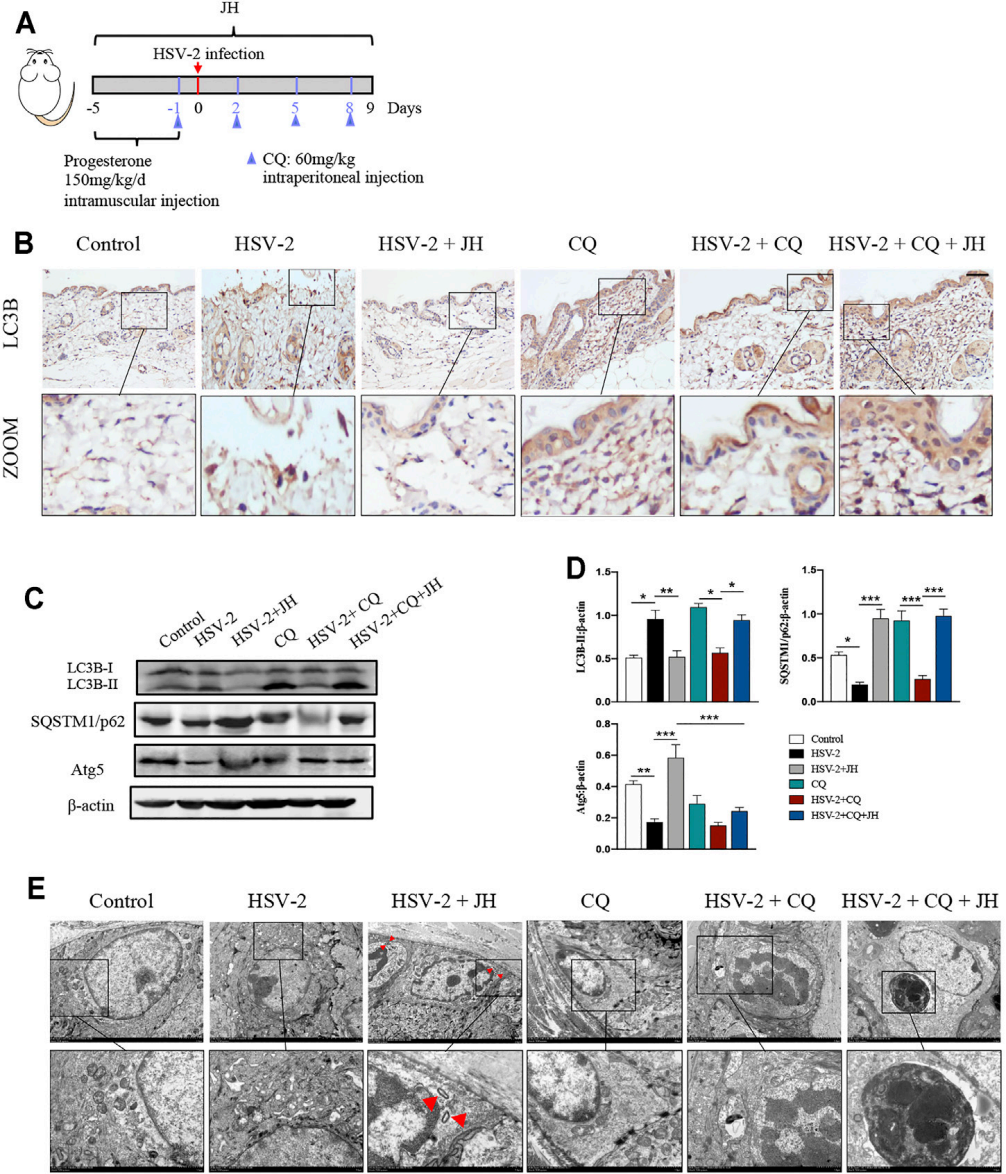
HSV-2 infection leads to a blockage of autophagic flux *in vivo* and JZ-1 administration induces autophagy in HSV-2-induced GH. **(A)** The protocol of administration of CQ in animal experiment. **(B)** Representative immunohistochemistry staining for LC3B in vulva of different groups. **(C)** Representative western blot for LC3B-II, SQSTM1/p62 and Atg5 protein expressions in vulva. **(D)** The quantification of LC3B-II, SQSTM1/p62 and Atg5 western blot. **(E)** TEM of different groups in vulva (the red arrow indicates the autophagy structure). All data are presented as means ± SEM. *p* < 0.01 (**), and *p* < 0.001 (***); (*n* = 6).

Our previous work has confirmed that HSV-2 infection can inhibit autophagy flux *in vitro*, but it is unclear whether HSV-2 infection also blocks autophagic flux *in vivo*. Microtubule-associated protein 1 LC3B is an indicator of autophagic flux. LC3 is initially synthesized in an unprocessed form, proLC3, which is proteolytically processed into the cytosolic LC3B–I isoform, and is finally modified into the PE-conjugated form, LC3-II, when autophagy is induced. In addition to LC3, SQSTM1/p62 can also be used as protein marker, the SQSTM1 protein serves as a link between LC3 and ubiquitinated substrates. And Atg5 mediate phagophore expansion and autophagosome formation during autophagy induced (Klionsky et al., 2016). Usually, the expression level of LC3B-II is positively correlated with autophagy. However, the accumulation of LC3B-II also occurs when autophagy flux is blocked. Therefore, to elucidate the true effect of HSV-2 infection on autophagic flux *in vivo,* we applied the autophagy flux inhibitor chloroquine (CQ) in later investigations (Figure 5A). Inhibiting autophagy in advance by CQ allows us to intuitively observe the true influence of upstream factor on autophagic flux.

As shown in Figures 5B–D, the results showed that the expression of LC3B in model group was higher and the expression of SQSTM1/p62 decreased than those in control group, while JH treatment restored their expression. If only these three groups are set, we may draw a wrong conclusion that HSV-2 infection will increase autophagy, while JZ-1 will inhibit autophagy *in vivo.* Actually, after the application of CQ, we can learn the true influence of HSV-2 infection and JZ-1 on autophagic flux *in vivo.* Compared with CQ group, it was observed that the expression of LC3B and SQSTM1/p62 in HSV-2+CQ group were both reduced, and compared with HSV-2+CQ group, JH administration obviously increased LC3B and SQSTM1/p62 expression. In addition, the expression of Atg5 decreased in model group compared with control group, while JH treatment increased its expression significantly (Figures 5B–D). Consistently, the TEM results found that JH group showed more autophagy structures than HSV-2 group (Figure 5E). Altogether, these results revealed that HSV-2 infection blocks autophagic flux *in vivo*, while JZ-1 administration induces autophagy in GH.

### The Anti-Apoptotic Effect of JZ-1 is Mediated by its Induction of Autophagy

**FIGURE 6:**
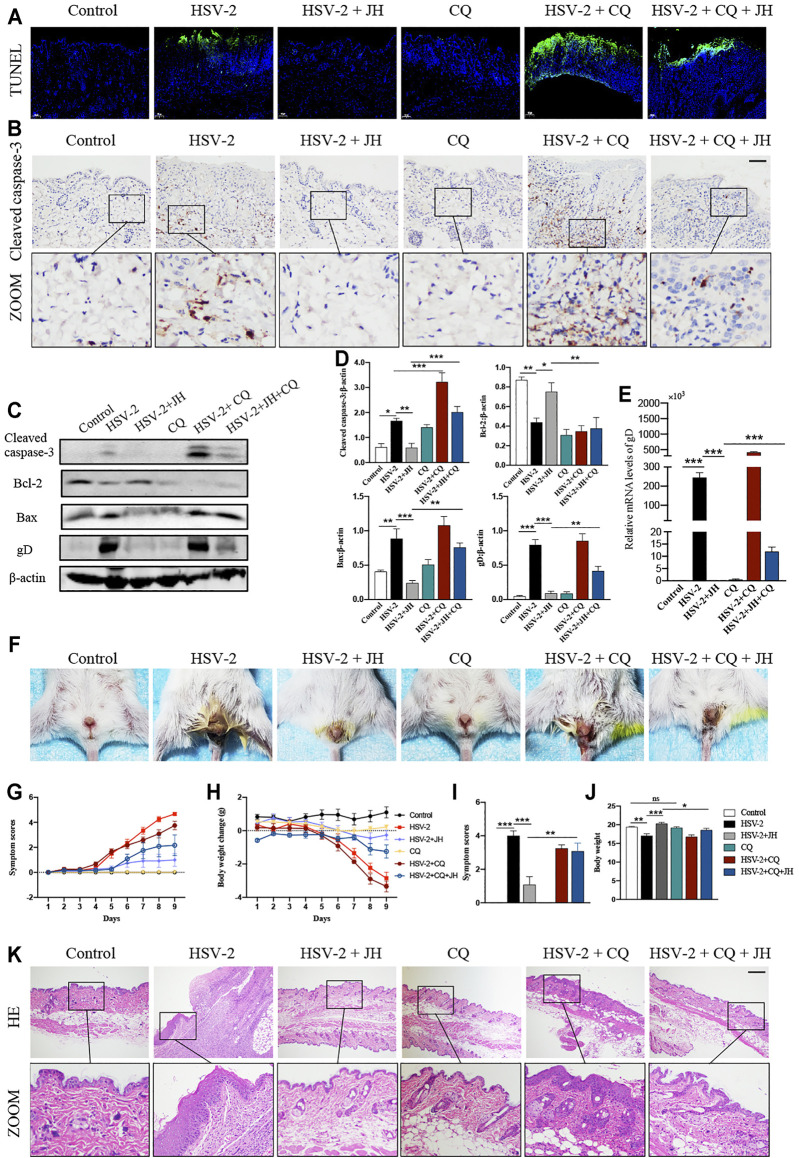
The anti-apoptotic effect of JZ-1 is mediated by its induction of autophagy. **(A)** Representative TUNEL staining in vulva of different groups. **(B)** Representative immunohistochemistry staining for cleaved caspase-3 in vulva of different groups. **(C)** Representative western blots for cleaved caspase-3, Bax and gD in vulva of different groups. **(D)** The quantification of cleaved caspase-3, Bax and gD western blots. **(E)** The mRNA levels of gD in vulva of different groups. **(F)** Pictures of the vulva of balb/c mice. **(G)** Symptom scores of different groups. **(H)** Body weight of different groups. **(I–J)** Statistical analysis of symptom scores ND Body weight. **(K)** Representative liver H&E staining of different groups. All data are presented as means ± SEM. *p* < 0.01 (**), and *p* < 0.001 (***); (*n* = 6).

According to above results, we have verified that HSV-2 infection can induce cell apoptosis and block autophagy flux *in vivo*, while JH can suppress cell apoptosis and induce autophagy flux. Since promoting autophagy has an inhibitory role on cell apoptosis during virus infection, thus we wonder whether the anti-apoptotic effect of JZ-1 is mediated by its induction of autophagy. Compared with CQ group, HSV-2+CQ group showed massive apoptotic cells. Meanwhile, it was also found that HSV-2+CQ group displayed more apoptotic cells than HSV-2 group (Figures 6A,B). These results suggested that the inhibition of autophagy is strongly associated with increased cell apoptosis, which was consistent with previous studies. As expected, the apoptosis level in HSV-2+CQ + JH group was significantly increased and the autophagy level was obviously decreased compared to HSV-2+JH group (Figures 6A,B and Figures 5B–D). These results were also supported by further western blots and qRT-PCR assay (Figures 6C–E). Taken together, our data demonstrated that the anti-apoptotic effect of JZ-1 can be mediated by its induction of autophagy.

Besides, it was observed that the degree of vulva ulceration in the mice of HSV-2+CQ and HSV-2 + CQ + JH group is also heavier than that of HSV-2 group and HSV-2 + JH group, respectively (Figure 6F), the symptom score is higher, and the weight loss is more significant (Figures 6G–J). The result of H&E staining showed the same trend (Figure 6K). This phenomenon is attributed to that the treatment of CQ inhibits the beneficial effects of autophagy and exacerbates cell apoptosis in GH.

### JZ-1 Inhibits PI3K/Akt/mTOR Pathway in HSV-2-Induced GH

**FIGURE 7:**
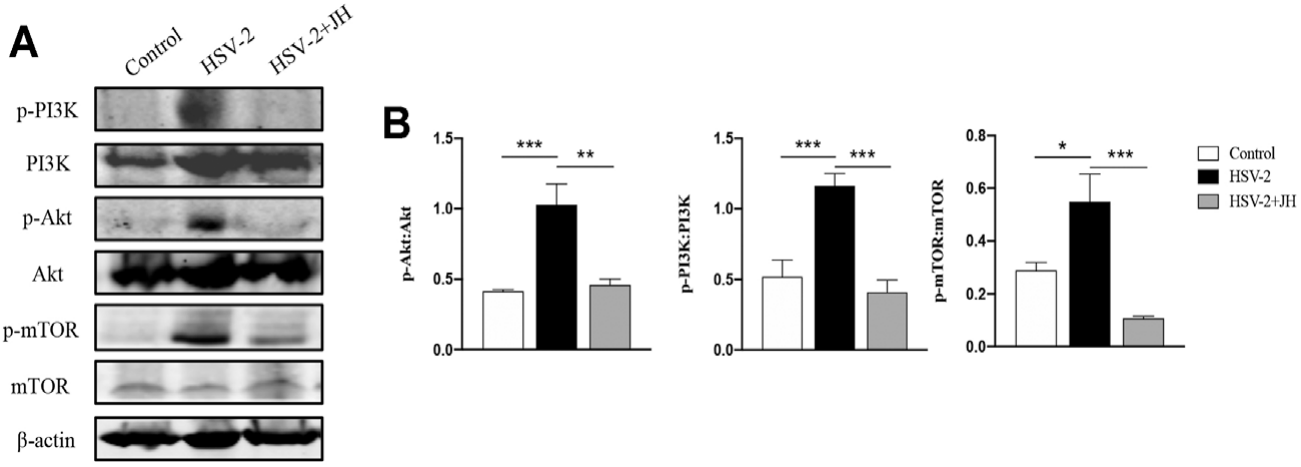
JZ-1 inhibits PI3K/Akt/mTOR pathway in HSV-2-induced GH. **(A)** Representative western blots for p-PI3K, PI3K, p-Akt, Akt, p-mTOR, and mTOR in vulva of different groups. **(B)** The quantification of p-PI3K, PI3K, p-Akt, Akt, p-mTOR, and mTOR western blots. All data are presented as means ± SEM. *p* < 0.01 (**), and *p* < 0.001 (***); (*n* = 6).

The mammalian kinase target of rapamycin (mTOR) is the main regulator of autophagy, which is regulated by classic PI3K/Akt signaling pathway (Manning and Cantley, 2007; Heras-Sandoval et al., 2014). In our previous study, we have shown that JZ-1 can induce autophagy *in vitro* via inhibiting PI3K/Akt/mTOR signaling axis to protect against HSV-2 infection. Therefore, we also detected this pathway in this *in vivo* study. The results showed that the phosphorylation level of PI3K, Akt, and mTOR in HSV-2 group were significantly increased compared to the control group, and JH administration restored the phosphorylation level of these proteins (Figures 7A,B). These results indicated that, similar to *in vitro* study, JZ-1 induces autophagy in HSV-2-induced GH and its underlying mechanisms may be associated with the inhibition of PI3K/Akt/mTOR pathway.

### JZ-1 can Exert its Anti-Apoptotic Effect by Inducing Autophagy in VK2/E6E7 Cells

**FIGURE 8:**
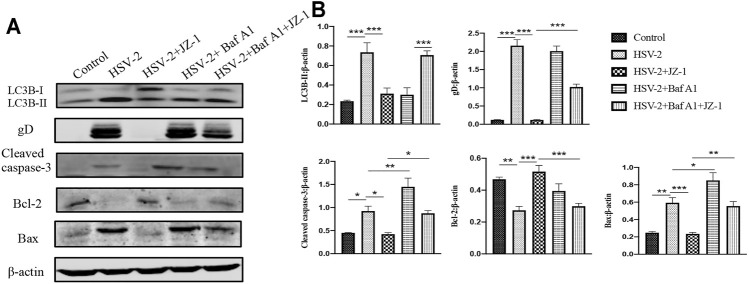
JZ-1 can exert its anti-apoptotic effect by inducing autophagy in VK2/E6E7 cells. **(A)** Representative western blots for LC3B-II, gD, cleaved caspase-3, and Bax in VK2/E6E7 cells of different groups. **(B)** The quantification of LC3B-II, gD, cleaved caspase-3, and Bax western blots. All data are presented as means ± SEM. *p* < 0.01 (**), and *p* < 0.001 (***); (*n* = 6).

As a supplement, we further verified the above mechanism on VK2/E6E7 cells. In the *in vitro* study, we applied bafilomycin A1 (Baf A1) as the inhibitor of autophagy flux. As shown in Figures 8A,B, the expression levels of cleaved caspase-3 and Bax significantly increased both in HSV-2 and HSV-2+Baf A1 groups, and JZ-1 administration obviously reduced the expression of above proteins and induced autophagy process. Meanwhile, the cell apoptosis level in HSV-2+Baf A1 and HSV-2+Baf A1+JZ-1 group were significantly higher than those in HSV-2 and HSV-2+JZ-1 group, respectively. These results demonstrated that JZ-1 can exert its anti-apoptotic effect by inducing autophagy in VK2/E6E7 cells, and the inhibition of autophagy impairs its anti-apoptotic effect.

## Discussion

In recent years, although the prevalence of genital herpes caused by HSV-1 has increased (Looker et al., 2015), but compared with HSV-2, HSV-1 is much less likely cause recurrent genital infections (Engelberg et al., 2003). HSV-2 infection still accounts for the vast majority of global genital herpes diseases, about 95% (Looker et al., 2020). The typical symptoms of genital herpes are frequent, painful, recurrent genital lesions, and are accompanied by many social and psychological distress (Gupta et al., 2007). In addition to the characteristics of recurrence, the reasons why it is hard to control HSV-2 infection may include: 1) GH caused by HSV-2 infection is transmitted by sexual activities, and the preventive strategies are not strictly adopted worldwide. 2) Current drug treatments are not satisfactory, and some people are resistant to the nucleoside analogues. 3) There is no breakthrough in vaccine research. 4) Additionally, the research on the mechanism of HSV-2 infection is also lacking at present.

The therapeutic effects of TCM on infectious diseases has been verified in multiple public health incidents. Chinese medicine has played a critical role in treating whether the severe acute respiratory syndrome (SARS) in 2003 or the coronavirus disease 2019 (COVID-19). Similarly, traditional Chinese medicine and its active ingredients have been proven to have significant anti-herpes simplex virus effects (Li et al., 2018; Shen et al., 2019; Feng et al., 2020). JZ-1 is an in-hospital preparation that has been used clinically for many years. It has a good effect on common female lower genital tract infections such as vaginitis and cervicitis. Clinical and experimental studies have shown that JZ-1 can significantly improve the symptoms of vaginal congestion, cervical erosion, abnormal vaginal discharge, vulvar itching and frequent urination caused by *U. urealyticum* infection (Hong et al., 2007), and can prevent *C. albicans* and *T. vaginalis* infections *in vivo* and *in vitro* (Chen et al., 2009a; Chen et al., 2009b; Chen et al., 2009c). Our previous *in vitro* experiments have also confirmed that the Chinese medicine JZ-1 has a significant anti-HSV-2 infection effect (Shao et al., 2020). However, it is unclear whether JZ-1 can protect against HSV-2 infection *in vivo*, and the underlying mechanisms for JZ-1 also needs to be clarified. In this present study, we confirmed the *in vivo* anti-HSV-2 infection effect of JZ-1, and revealed that JZ-1 exerts the anti-HSV-2 infection effect by inhibiting cell apoptosis via inducing autophagy.

Apoptosis and autophagy are the most common and widely studied types of programmed cell death. It is well-known that there is a complex functional relationship between apoptosis and autophagy. Generally, autophagy constitutes a stress adaptation that avoids cell death (inhibit apoptosis), whereas under certain circumstances, it constitutes an alternative cell-death pathway (promote apoptosis). In this study, we found that HSV-2 infection induced cell apoptosis, and JZ-1 can exhibit the anti-viral effect by inhibiting apoptosis both *in vivo* and *in vitro*. From our previous study, we learned that HSV-2 infection could inhibit the autophagy flux *in vitro*, which is conducive to the virus evading cell phagocytosis. Hence, we planned to investigate the effects of JZ-1 on autophagy flux *in vivo*. We found that in many studies, it is unscientific that only use the changes of the key autophagy protein LC3B to draw a conclusion about autophagy flux changes. According to the latest edition of the autophagy monitoring guidelines (Klionsky et al., 2016), a variety of methods for monitoring autophagy flux are recommended, but it is clearly stated that the conclusion that autophagy flux is induced or inhibited cannot be drawn based on a single method. In previous *in vitro* experiments, we chose autophagy flux inhibitor bafilomycin A1 to verify the true effect of HSV-2 on autophagy flux. Similarly, in this study, we used CQ, another autophagy flux inhibitor, to explore the true effect of HSV-2 infection on autophagy flux in balb/c mice. Autophagosomes need to be fused with lysosomes to form autolysosomes to perform their functions after forming, and autophagy flux would be blocked if autophagosomes cannot fuse with lysosomes, which means that autophagy is inhibited. The role of CQ is to hinder the fusion of autophagosomes and lysosomes (Mauthe et al., 2018). In this study, the expression level of LC3B-II increases in vulva of HSV-2 infected balb/c mice, which is consistent with the performance of autophagy being induced under normal circumstances, but we cannot conclude that HSV-2 induces autophagy only based on the changes of LC3B-II. According to our results, after the application of CQ, compared with CQ group, the expression of LC3B-II in HSV-2+CQ group did not further increase, but decreased; and the expression of LC3B-II in HSV-2+CQ + JH group significantly increased compared to HSV-2+CQ group, which indicated that HSV-2 infection blocked autophagy flux, and JH administration induced autophagy flux. Therefore, at least during HSV-2 infection, we demonstrated that promoting autophagy inhibits cell apoptosis. Additionally, we suggested that in the study of autophagy, relevant compounds and various methods should be used rationally, and conclusions should be made cautiously to ensure the authenticity and scientificity.

Next, we explored the underlying mechanism that regulates autophagy under JZ-1 treatment. The PI3K/Akt/mTOR axis is well-recognized as an important signaling pathway for regulating autophagy, which has been confirmed in our previous *in vitro* experiments. PI3K/Akt/mTOR signal pathway consisting of two parts: phosphatidylinositol 3-kinase (PI3K) and its downstream molecule serine/threonine protein kinase B (PKB; also known as Akt) (Xu et al., 2020), which plays a vital role in many cellular processes essential for homeostasis, including the cell cycle, cell survival, cell growth and proliferation, inflammation, metabolism, and apoptosis (Sun et al., 2020). In this study, we detected the phosphorylation levels of PI3K, Akt and mTOR, and found that HSV-2 infection induces the activation of this pathway, while JH inhibits this pathway. These results indicated that the promotive effect of JZ-1 on autophagy may be mediated by the PI3K/Akt/mTOR signaling pathway.

## Conclusion

**FIGURE 9:**
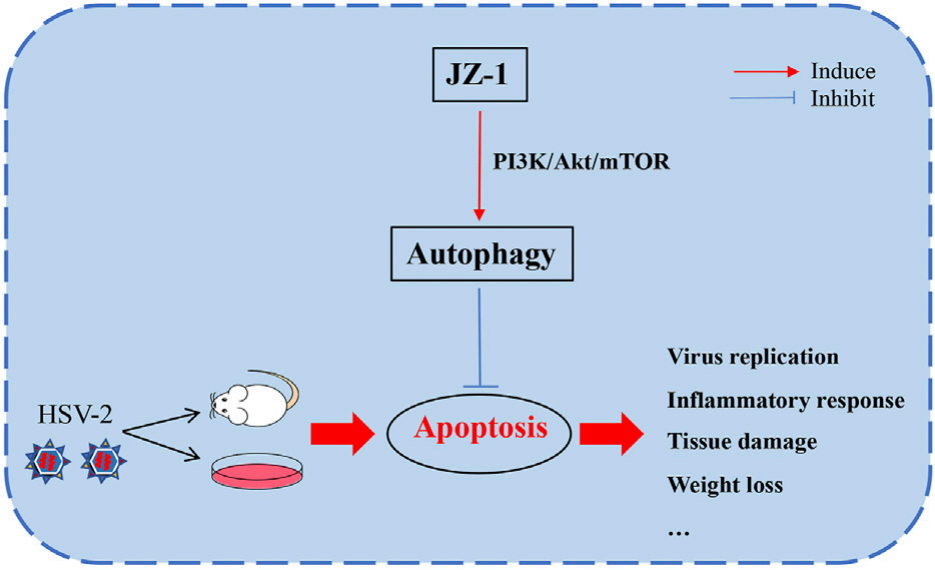
JZ-1 alleviates HSV-2 infection-induced genital herpes in balb/c mice by inhibiting cell apoptosis via inducing autophagy. In the pathogenesis of GH, HSV-2 infection leads to massive cell apoptosis, which largely promote the progression of disease. JZ-1 administration can alleviate HSV-2 infection-induced GH in balb/c mice by inhibiting cell apoptosis via inducing autophagy, and the underlying mechanisms may be associated with the inhibition of PI3K/Akt/mTOR pathway.

In conclusion, this study explored the potential effects and underlying mechanisms of JZ-1 on HSV-2 infection-induced genital herpes in balb/c mice. Our data demonstrated that JZ-1 can alleviate HSV-2 infection-induced GH in balb/c mice by inhibiting cell apoptosis via inducing autophagy, and the underlying mechanisms may be associated with the inhibition of PI3K/Akt/mTOR pathway (Figure 9).

## Data Availability

The original contributions presented in the study are included in the article, further inquiries can be directed to the corresponding author.

## Abbreviations

JZ-1, JieZe-1; JH, high-dose JZ-1 gel; GH, genital herpes; HSV-2, herpes simplex virus type-2; TEM, transmission electron microscope; HIV, human immunodeficiency virus; TUNEL, terminal deoxynucleotidyl transferase (TdT)-mediated dUTP nick-end labeling; Baf A1, Bafilomycin A1; CQ, chloroquine; PI3K, phosphoinositide 3-kinase; mTOR, mammalian target of rapamycin; IL-6, interleukin 6; IFN, interferon; TNF, tumor necrosis factor; LC3, microtubule-associated protein 1 light chain 3(MAP1LC3/LC3); Atg, autophagy-related; gD, glycoprotein D; H&E, hematoxylin-eosin; TCM, traditional Chinese medicine.
